# Professional Education and Training in Clinical Sexology in Europe

**DOI:** 10.1080/19317611.2026.2713157

**Published:** 2026-08-17

**Authors:** Elsa Almås, Alain Giami, Osmo Kontula

**Affiliations:** a Clinical Psychology, and Clinical Sexology (NACS), Ordbruk as, Grimstad, Norway, University of Agder, Grimstad, Norway; b INSERM, Paris, France; c Population Research Institute, Helsinki, Finland

**Keywords:** Clinical sexology, sex therapy, sexual health, professional education, political recognition

## Abstract

**Objectives:**

The aim of this study was to describe characteristics of professional education programs in clinical sexology, including organizers, academic affiliations, target groups, program levels, admission criteria, and jurisdictional recognition of sexology as a profession in Europe. This data will also improve our understanding of how sexology is used as a tool in education of counselors and therapists, and how sexology can be used to enhance sexual health.

**Methods:**

In 2023, the WAS committee for Professional Education and Training in Clinical Sexology (WAS PES) conducted an international survey of educational programs. An inductive approach was used to generate data about educational programs worldwide. A structured questionnaire was designed in collaboration with the University of Agder and distributed digitally to WAS organizational and individual members. Initial recruitment occurred via the WAS newsletter, with subsequent participant recruitment leveraging snowball sampling to include programs that had not responded to initial recruitment efforts.

**Results:**

The survey yielded 77 responses from 18 European countries. After excluding duplicates, non-clinical entries, and irrelevant submissions, 48 programs underwent further analysis. These included 10 full master’s degrees, 14 university-affiliated master’s-level courses, two sexological counseling certifications, 14 continuing education programs in psycho-sexual therapy, and eight short-term training courses.

**Conclusion:**

The proliferation of clinical sexology training programs underscores the need for curricula that align with both national standards and practitioner competencies. While the European region offers diverse educational pathways, there is a critical need to standardize accreditation frameworks and clinical guidelines at the European level. The World Association for Sexual Health (WAS), in partnership with the European Federation for Sexology (EFS), European Society for Sexual Medicine (ESSM), and national societies, is positioned to lead this standardization effort in line with the Porto Proclamation of 2025.

## Introduction

In 1972, a World Health Organization (WHO) consultation highlighted systemic gaps in opportunities for health practitioners to study human sexuality and a critical shortage of educators to develop training programs. A subsequent 1974 meeting led to a Technical Report (WHO, 1975) outlining recommendations for integrating sexology into academic curricula. The report emphasized that “(…) *education, counseling, and therapy are inseparable parts of sexual health care, and (…) education of the community has the priority in being able to affect positively the greatest number of people. There is a great and universal need to bring about positive change in the existing attitudes toward human sexuality among the general public, as well as among health and other personnel who are responsible for sex education and sex counseling*” (Abraham & Pasini, 1975; Haeberle & Gindorf, 1993).

Despite incremental progress in sexological research and the establishment of professional organizations globally, the WHO’s 1975 recommendations remain inadequately implemented (Giami, 2002; Kirana & Tripodi, 2025; Verrastro et al., 2020). In 1993, German sexologist Rolf Gindorf noted the scarcity of academic opportunities in the field, stating: “*… the prospective students of sexology would be hard put to find academic education and training*.” During what Gindorf describes as “*the political reform years of the seventies, three full professors for sexology were appointed for three German universities: Frankfurt, Kiel and Hamburg. Full professors of sexology are still a rarity in Europe*.” Gindorf writes laconically: “*To these three surviving sexology professors, add a few other university professors with sexological activities who are officially disguised as ‘plain’ physicians, sociologists, psychologists, educators, social workers, philosophers, or other.*” (Haeberle & Gindorf, 1993, p. 28).

In this article, the definition of sexology that was proposed by Erwin Haeberle and Rolf Gindorf in 1993 is used: “*Sexology shall mean that field of science dealing systematically with human sexuality: asking questions and trying to answer them in certain logical and methodological (‘scientific’) procedures*.” (Haeberle & Gindorf, 1993).

Since 2000, sexology in Europe has changed from a primarily medical field to an interdisciplinary field, including psychologists, psychotherapists, nurses, social workers, and physiotherapists as clinical actors, as well as teachers, lawyers, sociologists and activists (Almaas & Giami, 2006; Giami & de Colomby, 2003, 2006; Giami & Michaels, 2020; Kontula & Valkama, 2000; Simonelli et al., 2006). This has included a range of educational programs, from those targeted toward health professionals with bachelor degrees as basic professional education (Fugl-Meyer et al., 2001), to those meant for medical doctors and psychologists (Simonelli et al., 2010).

A survey from France shows that the proportion of women in sexology had increased to 83%, and 67% of sexologists were non-physicians. While 62% of sexologists have a degree in sexology or sexual health, most do not dedicate more than 50% of their professional time to sexology. Physicians continue to have high visibility relative to other professional disciplines (e.g. in congress‐speaking etc.) (Giami & Michaels, 2020).

In the early 2000s, Kontula (2011) conducted a European survey of existing professional trainings in sexology. Information of sexological trainings from 25 European countries was collected. Kontula inductively constructed 6 types of training in sexology in Europe: (1) a medical model, (2) a clinical model integrating medical and psychological approaches, (3) separate education in clinical sexology and human sexology, (4) sex therapy model, (5) human sexuality model, and (6) the Nordic human sexology model (Kontula, 2011).

Still, clinical sexology remains absent from core curricula in most European healthcare training programs. Even in disciplines such as urology, psychiatry, psychology, and gynecology, sexology is typically limited to brief introductory modules rather than comprehensive programs (Wylie & Weerakoon, 2010).

## Objectives

The aim of this study was to systematically identify and describe training programs, affiliations (universities, non-governmental organizations (NGO’s), and private institutions), academic professions involved, target groups, academic levels and scope of the programs, clinical level according to the PLISSIT–model, supervision, and jurisdictional recognition of sexology as a profession in the country in which the program is located.

The PLISSIT framework outlines a tiered approach to addressing sexual health concerns: Permission to talk about sexuality (P), Limited Information (LI), Specific Suggestions (SS), and Intensive Therapy (IT). Expertise in clinical sexology (medicine or psychology) are only necessary for the most complicated cases, which is a minor part of the totality of the professional practice (Annon, 1976). According to the Bio-Psycho-Social model (Engel, 1977a), expertise relevant to the PLISSIT model may be categorized as *biologica*l (e.g., medicine, endocrinology, surgery, psychiatry, rheumatology, oncosexology, disabilities, chronic disease), *psychologica*l (e.g., human development, communication skills, relational issues, sexual trauma, psychotherapy, psychoanalysis), and *social* (e.g., cultural influences, sexual behavior, violence and abuse, forensic sexology, rehabilitation). These domains of expertise are applicable across all levels of the PLISSIT model.

## Material and method

An inductive approach was used to generate data about educational programs worldwide. A cross-sectional survey was designed, including a combination of closed- and open-ended questions about the characteristics of programs focusing on clinical sexology. The primary aim of the questionnaire was to capture information from stakeholders actively engaged in professional education in clinical sexology. Single items were replicated and adopted from previous surveys that were developed for the same target group of sexology professionals (Kontula, 2011; Kontula & Valkama, 2000). This approach enabled an update to the previous research in this area, describing both the current situation and the evolution of practices and programs in professional trainings for clinical sexologists. Additionally, the survey assessed participants’ perceptions of the societal relevance of clinical sexology training and governmental recognition of sexology as a profession.

The questionnaire was distributed electronically through the WAS network via newsletter and social media posts. Recipients included member organizations, individual members, and affiliates. Recruitment followed a purposive, non-probability sampling strategy. The sample was intentionally non-representative since the reference population of programs was not known at the date of the survey. No attempt was made to recruit a random or proportionally representative sample of all sexological/sexual health organizations globally.

The global study organized responses through the six regional federations of the World Association for Sexual Health (WAS):

- Sexual Health Africa (SHA)
- Asia-Oceania Federation for Sexology (AOFS)
- Eastern Mediterranean Federation for Sexual Health (EMFeSH)
- European Federation of Sexology (EFS)
- Latin American Federation of Sexology and Sex Education Societies (FLASSES)
- North American Federation for Sexual Health (NAFSH)

Given significant regional variations, each federation conducted context-specific analyses aligned with their cultural frameworks alongside standardized WAS Professional Education in Sexology (PES) protocols, based on the questionnaire.

Non-clinical programs explicitly targeting sexuality educators (e.g., teachers) or sexual health promoters (e.g., policymakers, legal professionals, journalists), were excluded. Programs on education in *basic sexology were included, as* these programs are basic for clinical sexology, while also addressing other areas of sexology: sexuality education, sexual health promotion and research. As such, non-clinical groups may be included on this level.

Follow-up recruitment was done in each region based on a snowball sampling approach. Participation was voluntary, and no incentives were offered. Respondents could skip any items they preferred not to answer. The survey was based on the online web platform SurveyXactTM, which respondents accessed through a link. The survey was hosted by the University of Agder, Norway, and data presented were collected March–December 2023.

The subsequent sections focus on the European findings.

Data cleaning involved removing duplicates, non-European programs, and sexuality education curricula. For instance, Icelandic respondents trained at Australia’s Curtin University were excluded. Qualitative analysis was employed due to insufficient sample sizes for meaningful statistical inference.

In Europe, the Bologna Process serves as the standardized framework for academic credit allocation, where one academic year equates to 60 ECTS credits, representing 1,620 total workload hours. This distribution comprises 25% instructor-led contact hours, 25% collaborative group work, and 50% independent study. Each ECTS credit corresponds to 27 hours of student effort. For programs outside universities, the scope of the program may be described by teaching hours only. Therefore, the comparison of university and non-university programs is challenging, as they are not organized according to the same rules.

## European results

From an initial pool of 77 responses, 48 submissions across 18 countries met inclusion criteria.

## Organizers and academic levels

There is great diversity of programs that are categorized as shown in Table 1:

**Table 1.** Diversity of education and training.

	Master’s degree (160-120 ECTS)	Master level (5-60 ECTS)	Counseling NGO/private	Psycho-sexual NGO/private	Short courses
Belgium	1				2
Croatia				1	
Denmark		2	1	1	
Finland		2	1	1	
France	1				
Germany	1			2	1
Greece					1
Ireland		1			
Italy		1		2	
Kosovo				1	
Luxembourg					1
Netherland				2	1
Norway	1	1		3	1
Portugal	2			1	
Spain	2	7^1^			
Sweden	1				
Turkey				1	
UK	1				
ESSM					1
**Total**	**10**	**14**	**2**	**14**	**8**

Clinical sexology is taught both at the master’s level at universities and as continuing education in private institutions and national sexological societies. The level and scope of the programs are highly variable. The included programs have all been qualified as clinical or highly clinically relevant, and have been organized by five categories:

- 10 master’s programs at universities (120 ECTS, corresponding to 2 years academic work)
- 14 university courses at master’s level (60 ECTS or less, corresponding to 1-year academic work or less)
- 2 sexological counseling programs (Private/NGO)
- 14 trainings in psycho-sexual therapies (Private/NGO)
- 8 short courses (Private/NGO)

While 3 doctoral-level program were identified in Europe during data collection, 2 were excluded from analysis due to lack of data.

The 24 sexology programs across *21 universities* included: KU Loeven (Belgium), University of Copenhagen, University of Aalborg (Denmark), Metropolia University of Applied Sciences in Helsinki, Tampere University of Applied Science (Finland), Toulouse University Jean Jaurés, University Hospital Dr. Peset (Spain), Medical School Hamburg (Germany), Dublin University (Ireland), University of Milano, Bicocca (Italy), Agder University (Norway), University of Lusófona, University of Porto (Portugal), University of Almeira, European University of Atlantic, University of Barcelona, University of Girona, National University of Distance Education, University of Nebrija, (Spain), Malmö University (Sweden), and University of Southampton (UK). Some of the university-based programs offer education in sexological counseling.

Continuing education programs outside universities frequently adopt the designation “*sexual psychotherapy*,” with titles such as “*Professional training in sexual psychotherapy*,” but there are also titles like “*Specialization course in clinical sexology*.”

The responses included 24 programs administered by sexological societies, private institutions, and NGOs: two counseling programs, 14 psychosexual therapy trainings, and eight short courses (summer-schools or brief workshops).

Program durations range from 24 hours to three years for counseling tracks; 16 hours (Sexual Attitudes Reassessment or SAR-course) to four years (3,900 hours) for psychosexual training; and 5–50 hours for short-term courses.

Primary participants of the programs include licensed physicians, psychologists, psychotherapists, counselors, nurses, and social workers. The programs function as continuing clinical sexology training for professionals, blending medicine, psychotherapy, nursing, physiotherapy, social work, pedagogy, and sexology.

Two *Sexological counseling programs* were explicitly reported: Denmark: Danish Association for Clinical Sexology; Finland: SEXPO.

*Fourteen Psychosexual* training programs responded to the survey. These postgraduate continuing education initiatives were typically administered by professional associations or independent institutes: *Croatia*, Croatian Association for Sexual Therapy: Professional training in sexual psychotherapy; *Denmark*, Institute for couple therapy: Professional training in sexual psychotherapy; *Finland*, School of Somatic sexology: Sexological bodywork & Somatic sex education; *Germany*, Munich, Institute for Sexual-, Psycho- und Traumatherapy: Certified training in sex therapy, sex counseling, sexuality and trauma; Nuremberg, Psychiatric Practice Nuremberg: Quality maintenance for mental health professionals regarding transsexualism; *Italy*, Florence: Institute for Research and Training: Sex education expert, sexology consultant, clinical sexologist; Rome, Institute for Clinical sexology, sexology consultant; *Kosovo*, Kosovo Body Psychotherapy Association – NOKTA: Psychosexual Psychotherapy; *Netherland*, RINO 1: Postgraduate sexology education; RINO 2: Course Consultant Sexual Health; *Norway*, Bufetat East: Family therapist/Sexological counselor; Bufetat South: Family therapist/Sexological counselor; *Portugal*, Sociedad Portuguesa de Sexologia Clinica: Specialization course in clinical sexology; *Turkey*, CISED: Sexual Therapy Training.

The eight *short courses* that were reported within this domain were:

*Belgium*, PXL University College of Applied Arts and Sciences: Summer school sexual health; Sensoa, Ghent: Good Lovers Flag System; *Germany*: Independent, affiliated with Sex Coach U: Sexual Attitude Reassessment; *Greece*, Hellenistic Psychiatric Association: Seminar for Gender Issues; *Luxembourg*, Institute for Family Planning: Post partum sexuality; *Netherland*, Zuidwester: Attention for sexuality in the support for people with intellectual disabilities; *Norway*, Norwegian Characteranalytical Institute: Continuing education in sexual health for licensed psychologists and psychiatrists; *Slovenia*, ESSM: Oxford Summer school in 2010.

### Academic affiliations

Academic affiliations refer to the professional field foundational to the educational curricula and organization of the programs.

Table 2 shows that most programs are grounded in interdisciplinary principles, integrating psychology, medicine, social work, pedagogy, nursing, psychotherapy, and physiotherapy.

**Table 2.** Professional or academic discipline. (several responses possible).

	Medicine	Psychology	Psycho-therapy	Nursing	Physio-therapy	Social work	Pedagogics/Education	Sexology	Other
Master Degree N = 10	5	6	3	3	2	3	3	8	2
Master level	8	7	6	6	4	3	3	9	1
Counseling	1	0	1	2	1	2	3	2	0
Psycho-sexual	8	8	11	2	1	2	3	11	Urology
Short courses	3	2	2	1	1	0	2	2	1
Total	25	23	23	14	9	10	14	32	5

Sexology is named by 67% (32/48) of programs, emerging as a dominant discipline alongside psychotherapy, medicine, and psychology, and serves as the primary academic foundation for most master’s programs in this study, constituting 80% (8/10) of Master′s degree offerings and 64% (9/14) of master’s level programs. Only University of Leuven (UL) reports to be an exclusively sexological program and offers sexological education from bachelor’s to doctoral level.

Seven programs explicitly identify sexology as their sole disciplinary focus: KU Leuven, Belgium, Finland’s School of Somatic Sexology (non-accredited), Germany’s SAR program (affiliated with Sex Coach U and Medical School Hamburg), Italy’s Institute of Clinical Sexology, and Spain’s Master of Sexology (Universities of Almería and Atlantic).

Sexology emerges as the *predominant* discipline university programs, followed by medicine and psychology. This trend extends to psychosexual programs outside universities, though psychotherapy replaces psychology as the secondary discipline alongside sexology.

All programs demonstrate a defined sexological identity, though clinical orientations vary.

Counseling programs and short courses are limited in quantity and span multiple disciplines. Certain short courses cater to distinct professional groups, such as physiotherapists, nurses, psychiatrists, or midwives, with curricula grounded in discipline-specific frameworks.

## Targeted groups and admission criteria

Interdisciplinarity is evident not only in the academic disciplines comprising the programs but also in the professional backgrounds of course participants across all categories.

Table 3 shows that primary target groups for university programs include medical doctors, psychologists, psychotherapists, and nurses.

**Table 3.** Target professional groups.

	GPs	Psychia-trists	Other medical specialist	Psycho-logists	Psycho-therap. Counsell.	Physio-therap.	Nurses	Social work	Teach.	Acti-vists	Other
Master Degree N = 10	7	7	7	8	7	5	7	7	5	3	4
Master Level N = 14	10	8	6	8	6	3	7	3	3	1	4
Counsel. N = 2	1	1	1	1	2	2	3	3	1	1	1
Pycho-Sexual N = 14	6	7	5	14	13	6	8	6	5	2	3
Short Courses N = 6	4	5	3	3	3	4	4	2	3	3	4
Total	28	28	22	34	30	20	29	21	17	10	16

A core admission requirement for all programs, except University of Copenhagen (for medical students), is holding an academic degree and/or clinical licensure, positioning sexology training as postgraduate specialization for practitioners seeking expertise in the field.

Psychosexual programs outside universities appear predominantly tailored to medical doctors, psychologists, and psychotherapists. The five most targeted professions are psychologists; psychotherapists/counselors; nurses; general practitioners; and psychiatrists.

Social workers, teachers and activists are included as the target students in some programs.

Counseling programs and short courses are fewer in number, and overall, evenly target the range of professional groups assessed. Some of these programs have specific professional groups and clinical problems as their focus.

*Sexology* remains a predominant label for academic and professional groups, with most university programs explicitly incorporating the term. Conversely, continuing education initiatives outside universities more commonly adopt descriptors like “*sexual psychotherapy*,” particularly for mental health practitioners, though “*sexual health*” occasionally appears interchangeably.

Specialized tracks increasingly address niche areas such as gender incongruence, postpartum-sexuality, trauma-informed care, adaptations for intellectual and cognitive disabilities, and systemic couple therapy.

## Interpreting the results through the PLISSIT-model

The responses to the questionnaire were interpreted through the PLISSIT model which proposes a segmentation and a hierarchization of the surveyed programs. Using the PLISSIT-model as an analytical tool, participants endorsed heterogeneous familiarity with the model. Within the European cohort (n = 48), 20 respondents (41.7%) provided actionable data regarding their implementation of tiers within the PLISSIT framework (Table 4).

**Table 4.** Level on the PLISSIT model.

ESSM Summer School	P	Short course
Lusufona, Portugal	P	Master’s degree
PXL, Belgium	P/LI	Short course
Copenhagen, Denmark	P/LI	Master level
Hamburg, Germany	P/LI	Master’s degree
Malmö, Sweden	P/LI	Master’s degree
Sexpol 2, Spain	P/LI	Master level
Luxembourg	LI	Short course
Oslo, Norway	IT	Short course
Loeven, Belgium	P/LI/SS	Master′s degree
Aalborg, Denmark	P/LI/SS	Master level
Agder, Norway	P/LI/SS	Master’s degree
Agder, Norway	P/LI/SS	Master level
Sexpol 3, Spain	P/LI/SS	Master level
Bufetat South, Norway	P/LI/SS	Sexual psychotherapy
Florence, Italy	P/SS/IT	Sexual psychotherapy
Sensoa, Belgium	P/LI/SS/IT	Short course
Valencia, Spain	P/LL/SS/IT	Master’s degree
Portugal, Porto	P/LL/SS/IT	Master’s degree
Sexpol 1, Spain	P/LI/SS/IT	Master level (Sex therapist)

The PLISSIT-model seems well known and acknowledged as a qualifier of educational programs. Four programs (Italy, Belgium, Spain and Portugal) offer education on the IT-level. Six programs (Belgium, Denmark, Norway and Spain) offer education on the SS-level.

**Table 6.** Recognition of sexologists in your country.

In your country, are…	*…there occupational positions labeled as “Sexologists”?* *Yes, in health care or educational* institutions:	*…. sexology/sexual health professional educational programs recognized as a “registered official profession by the government?*	*clinical sexologists or sexual experts recognized as a profession?*
Belgium	Yes	Yes	
Croatia			
Denmark	Yes		Yes
Finland	Yes		Yes
France	Yes		
Germany	Yes		Yes
Greece	Yes		
Ireland			
Italy	Yes		Yes
Kosovo	Yes	Yes	
Luxembourg			
Netherland	Yes	Yes	Yes
Norway	Yes	Yes	Yes
Portugal	Yes	Yes	Yes
Spain	Yes		Yes
Sweden	Yes		Yes
Turkey			Yes
UK			
Slovenia ESSM		Yes	
Total	13 countries	6 countries	10 countries

Only two of the psychosexual programs (Norway and Italy) report the level of their programs on the PLISSIT-model, and only one of the programs (Italy) is on the IT level. Programs offering master′s degree or master’s level courses endorsed the full range of PLISSIT-model levels.

## Supervision

Significant variability exists in supervision requirements across programs examined in this study. This study identified disparities in supervision hours:

- Master’s programs: Only 1/10 requires supervised training (35 hours).
- Master’s-level programs: 6/14 require 12–100 supervision hours.
- Counseling programs: 2/3 require 16–75 supervision hours.
- Psychosexual therapy programs: 11/15 require 24–224 supervision hours.
- Short courses: Only the ESSM Summer School requires 10 supervision hours.

Notably, many university programs lack integrated supervision frameworks. For example, Nordic countries adhere to a common accreditation system through the Nordic Association for Clinical Sexology (NACS). Students must independently secure external supervision as required by NACS.

While most programs emphasize clinical training, only one master’s program (University of Girona) and six of 14 master’s-level programs mandate supervised practice. This suggests a predominant theoretical/academic orientation in university curricula, with institutions avoiding the logistical and financial burdens of supervision for their candidates. Alternatively, this could indicate a scientific orientation which does not consider issues related to the subjective implication of the sexologist and its impact on practice.

Eleven of the 15 Psychosexual therapy programs offer supervision, indicating that these are more clinically oriented.

One of eight short courses require supervision. This may be due to the short format of the programs, and a primary focus on theoretical training within their short scope.

## Accreditation

For education programs to be formally acknowledged and recognized, accreditation is an important standard to measure and ensure quality of training.

Table 5 shows that university programs are accredited according to their academic standing. Variability exists in the application of the “master’s” designation, with some institutions awarding master’s degrees for 60 ECTS programs, whereas the Bologna model in Europe mandates 90–120 ECTS for full master’s degrees.

**Table 5.** Completion to training program.

	ECTS	Scope	No answer	Master degree	PhD	Diploma/Certificate	Other	No
Master Degree N = 10	10 (120 ECTS)			10	1			
Master level N = 14	14 (5–60 ECTS)		1	7	1	5		
Counseling Private N = 2		1 yr (800 h)				2		
Psycho-sexual N = 114		60 hr–5 yrs				10	2	2
Short courses private N = 8		5 h–50 h				6	1	1

The two counseling programs give a diploma for completing of the courses.

Psychosexual programs endorsed significant scope variation, ranging from 60-hours to 5-year curricula. Of the 14 programs analyzed, 10 confer diplomas aligned with their respective structures.

Of the eight short courses, six programs give a certificate for completing of the course.

## Jurisdictional recognition of sexology as a profession

Sexological or sexual health related positions in healthcare or educational institutions are reported in thirteen countries, as shown in Table 6. However, responses from only six European countries confirmed that Sexology/Sexual Health professional educational programs hold government-recognized professional status. While thirty-seven respondents reported a perceived need for clinical sexology or sexual medicine training programs, clinical sexologists or sexual health experts are reported to be formally recognized as professionals in only ten countries.

## Discussion

A few earlier studies have examined sexological education in Europe, including the 1975 WHO report (WHO, 1975), Haeberle & Gindorf, 1993 analysis in Sexology Today (Haeberle & Gindorf, 1993), Porto’s 2006 work (Porto, 2006b), the Euro-Sexo study by Giami et al. (Alcarcão et al., 2017; Almaas & Giami, 2006; Fugl-Meyer & Giami, 2006; Giami & de Colomby, 2006; Hurtado & Giami, 2017; Kontula & Valkama, 2000; Kristensen & Giami, 2006; Simonelli et al., 2006; Wylie, 2006), and Kontula’s 2011 survey on sexology training (Kontula, 2011).

While the medical model historically dominated sexological training paradigms, its influence has diminished in contemporary curricula. Physicians now constitute only one segment among multiple target audiences for most programs.

Since 2005, the concept of *sexual health* has come to replace *sexology* as a title for WAS, and some of the regional federations. This development must still be a subject for discussion. On the one hand, *sexual health* may be an easier concept about which to engage administrative and political authorities, while on the other hand it can be argued that *sexology* is the tool for our work while *sexual health* for all is the ideal aim for our work.

The Euro-Sexo study highlighted a disparity between the abundance of sexual health information and the practical application of treatments (Porto, 2006a). It further identified critical deficiencies in training programs, particularly in prevention strategies and research methodology, underscoring the need for systemic improvements.

By 2011, 25 countries offered such programs (Kontula, 2011). In 2011, the predominant model for sexological education involved training delivered by national associations and private institutes, as observed in 17 countries. Evidence indicates that universities played a limited role in postgraduate sexology training prior to the 2000s, representing a significant institutional gap (Haeberle & Gindorf, 1993). Since the early 2000s, significant advancements have occurred, with a notable increase in master’s programs (120 ECTS) across Europe. Analysis reveals dual educational pathways: 24 university-based programs and 24 initiatives administered by private entities or NGOs. Universities primarily deliver post-graduate level training targeting licensed healthcare practitioners.

Limited overlap between Kontula’s (2011) survey (54 programs across 25 countries) and the present WAS PES investigation (48 programs from 18 nations) may reflect program discontinuations and temporal gaps in data collection. Sixteen programs appear in both studies.

Sexological training in Europe primarily targets professionals with prior primary qualifications in another discipline, typically through complementary postgraduate programs. However, sexual health topics are still insufficiently integrated into foundational and primary curricula for healthcare personnel (Cesnik et al., 2017; Verrastro et al., 2020).

Of the 48 programs surveyed, 32 are grounded in sexology as either a professional or academic discipline. Other prominent disciplinary foundations include medicine and psychology.

A sense of sexological identity seems to be evident in the responses. Sexology is not a static field, but there are some characteristics that appear both over time and across countries and regions. One such characteristic is the bio-psycho-social approach (Almaas & Landmark, 2010; Engel, 1977b, 1980; Hartman & Fithian, 1974; Nimbi et al., 2021; Simonelli et al., 2010).

When the World Association for Sexology (WAS) was established at the Third International Congress for Medical Sexology in Rome in 1978, there was a clear demonstration of interdisciplinarity. The program included themes such as “Ethics and sexuality,” “Philosophical views on sex,” “Sociology and anthropology of sexual behavior,” as well as “Endocrinology and sex research,” “Sex therapy,” and “Sex education” (Forleo & Pasini, 1980). This interdisciplinarity has been present at all later WAS congresses.

A current and central debate revolves around whether sexology should exist as an independent academic discipline or function as a complementary specialization for existing professions (e.g., psychology, nursing, medicine). The latter approach risks relegating it to an informal adjunct role but is the dominant model of sexological education in Europe. This complementary specialization approach secures professional expertise in relevant fields and requires interdisciplinary cooperation.

*Clinical sexology* is that part of sexology that addresses sexual problems in individuals or couples through psycho-sexual therapy or sexological counseling. The clinical scope is commonly described by the tiered PLISSIT model, which includes four levels of intervention with increasing requirements for clinical skills: Granting permission (P) to be sexual, providing limited information (LI) about sexuality, offering specific suggestions (SS), and delivering intensive therapy (IT). Education and training in clinical sexology will implicitly or explicitly address these different levels of clinical approach.

*The Ex-PLISST model* recommends that the four levels of the model are used interchangeably, as all levels of the model may be useful at different stages of treatment. Thus, in advanced psychotherapeutic or medical work, it may be important to obtain *Permission* from individuals seeking care to share sensitive information across disciplines in order to provide effective treatment (Davis & Taylor, 2006; Taylor & Davis, 2007).

*Sexological counseling*, corresponding to the first three levels of the PLISSIT-model (permission, limited information, and specific suggestions), should be provided by trained professionals who meet clients at community levels: nurses, social workers, physiotherapists, and family or couples’ therapists with postgraduate training in sexology. Ideally, sexological counselors should be working in schools, community centers, healthcare facilities, childcare services, prisons, and hospitals. At this level, counselors are responsible for promoting sexual health and identifying cases that require referral to intensive therapy. Training in *sexological counseling* may be delivered by universities, national associations, or specialized institutes. These programs can extend up to two years, with in-person instruction commonly scheduled on weekends. Upon successful completion of all program requirements, including a final examination, participants are awarded a certificate by the respective educational provider.

*Sex therapy* is provided at the SS and IT levels of clinical expertise, based on P and LI. Sex therapy is generally understood as the treatment of defined sexual problems using a sexological approach, frequently based on the methods developed by Masters and Johnson (Masters & Johnson, 1966, 1970) and Helen Singer Kaplan (Kaplan, 1974). The approach continues to be elaborated on by other clinicians, like the introduction of mindfulness in sex therapy (Brotto et al., 2008). Sex therapy is most effective for individuals whose primary concern is sexual problems, often without significant psychological comorbidities. The conceptual boundaries of “sex therapy” require further delineation. Accreditation for sex therapists should define minimum professional standards.

*Psycho-sexual therapy* is primarily administered by psychologists and psychiatrists specializing in clinical sexology, also addressing underlying problems. In Italy (Rome), Croatia, Germany (Nuremberg), Netherland (RINO 1), Norway (BUF-Etat South), and Portugal (Portuguese Society for Clinical Sexology), admission to advanced psycho-sexual training requires licensure as a clinical psychologist, psychotherapist, or physician. These practitioners possess broad expertise beyond one single therapeutic approach, enabling them to employ tailored methodologies based on individual client needs. This can also include interdisciplinary work. Psycho-sexologists basically demonstrate advanced expertise in psychophysiology, neuropsychology, perceptual and learning mechanisms, complex trauma, chronic pain syndromes, and developmental-relational disorders and can address complex sexual problems (Kirana & Tripodi, 2025). Psychiatrists, with basic medical training, address psychiatric and somatic conditions impacting sexual health, including neurodiversity, pharmacological interventions, and adverse effects of medications.

*Medical sexologists* are physicians with sexology training who manage somatic contributors to sexual problems. They address iatrogenic sexual side effects of medications, employ nerve-sparing surgical protocols, and diagnose neuroendocrine pathologies that manifest with sexological symptoms or disrupt dyadic communication (Porst & Reisman, 2012).

*Sexual medicine* encompasses pharmacological management of sexual dysfunction, whereas *medical sexology* involves systematic evaluation of sexual comorbidities in systemic disease and treatment sequelae (Perelman, 2014).

Clinical guidelines emphasize that sexological treatment centers should integrate multidisciplinary teams or ensure clinicians maintain referral networks with complementary professionals (e.g., psychologists, urologists, or social workers).

There are several criteria that are important for education and training in clinical sexology to be acceptable tools to support clinical work with clients who seek help for problems concering sexuality and/or gender: Level of education (e.g., as described by the PLISSIT-model), supervision, and professional and jurisdictional accreditation.

The PLISSIT-model is not universally known or addressed by providers of sexological training but may be useful for identification and evaluation of the levels of intervention, training for people living with special needs, and for accreditation purposes.

The European results from the global survey highlight the need for international, regional, and national guidelines and accreditation. The current state of clinical sexology training is characterized by local initiatives that are based on each country’s understanding of sexology as a professional field, as opposed to a cohesive and comprehensive plan. Participation in national and international organizations (NACS, EFS, WAS) requires adherence to academic standards and ethical guidelines for membership.

In September 2025, the Porto Proclamation was signed by a coalition of thirty international organizations, civil society groups, professional societies, and academic leaders during the inaugural World Sexual Health Assembly in Porto^2^.

Of interest for professional education in sexology, are the following statements from the Porto Proclamation:

- *All sexual health-related interventions must be grounded in evidence that is rigorous and transparent, supported by research and informed by the lived experiences of people and communities.*
- *Accountability for sexual health and rights requires governments and institutions to be answerable through transparent laws and policies, adequate funding, effective monitoring, and meaningful community participation, particularly from marginalized groups.*
- *Every person has the right to accessible, respectful, and confidential sexual health services, with full informed consent and shared decision-making across the life course.*
- *Lasting progress in sexual health, rights, and justice requires equitable, multi-sector partnerships that place marginalized voices at the center and align efforts across health, education, training, justice, finance, and technology.*
- *… advancing priority levers and clear pathways of impact–driving legal reform, policymaking, community action, professional training, clinical services, …., and cross-sector collaboration at national, regional, and international levels.*
- *Promote and protect universal access to high-quality, integrated sexual health services, …., and professional training* (Edwards & Coleman, 2004; Giami, 2015; Giami et al., 2026).

This work supports and encourages staying up to date on the progress of sexology as a professional field by providing access to professional peer-reviewed journals, and opportunities to give and learn from presentations of scientific research and clinical experience at congresses.

The results from the WAS PES survey suggests that accreditation in clinical sexology may be meaningful to officially register titles such as *sexological counselor*, *sex therapist*, *psycho-sexual therapist,* or *medical sexologist*, each reflecting various levels or areas of training and clinical responsibility.

It may be time to develop international and regional guidelines for education and training in clinical sexology that can be adapted to the regional circumstances and differences that are demonstrated in this special issue. Both the bio-psycho-social and the PLISSIT-model can be of use for differentiating and tailoring training programs. The bio-psycho-social approach can be used on all levels of the PLISSIT-model, and it is applicable to both comprehensive programs directed to different professional groups (sexological counselors, sex therapists, psycho-sexual therapists, and medical sexologists) and shorter, problem-focused programs.

The absence of unified accreditation standards and terminology for sexology practitioners remains a critical barrier. There is yet some work to do in developing an international and regional system of accreditation, and this work should involve sexologists from different global, regional, and national sexological organizations. Standardized certification and accreditation processes for sexologists in Europe are critical to ensuring informed choice for patients, improving care quality, and fostering interdisciplinary collaboration. Multidisciplinary integration of sexual health curricula into medical and behavioral science programs could address these systemic gaps, but specific sexological specialist training programs, inside and outside universities are also needed. While the European Society for Sexual Medicine (ESSM) offers postgraduate training through its Oxford Summer School program – awarding a EU-recognized diploma – broader interdisciplinary consensus is lacking. A tripartite alliance between the World Association for Sexual Health (WAS), ESSM, and the European Federation for Sexology (EFS) together with the national sexological societies could establish evidence-based competency benchmarks and cross-border certification protocols.

Sexology has yet to achieve the political standing required to deliver comprehensive sexual healthcare for the European population. Persistent societal taboos surrounding human sexuality, coupled with insufficient policymaker and health administrator training, hinder progress. These barriers stem primarily from knowledge deficits rather than overt opposition, necessitating targeted educational initiatives for governmental stakeholders and health institutions.

Raising the level of professional education in the field of sexology and increasing the accessibility of sexological health care services will improve the professional appreciation of the field. It is in the common interest, and a benefit for all.

## Conclusions

Grounded in a strong sense of sexological identity, clinical sexology training programs in Europe exhibit significant heterogeneity in curricular structure and educational standards. Training programs must consider the needs of different stakeholders including (1) the scientific and ethical values of a professional community, (2) the requirements and expectations of customers and patients who need sexological treatment and counseling, (3) the inclusion in local (national) accreditation and health systems.

A robust, multidisciplinary pedagogical framework for European sexology training must prioritize the cultivation of professional competencies, including attitudinal readiness, evidence-based knowledge, practical skills and ethical adherence. Clinician biases or stigmatizing attitudes may impede therapeutic efficacy, necessitating explicit training in cultural humility and capacities toward self-reflexivity.

Furthermore, curricula should not only integrate current research on biological, psychological, and sociocultural dimensions of human sexuality to ensure up-to-date content. Programs should also include recent advances in pedagogical methods, utlizing approaches like workshops or group work fostering the sharing and analyzing of subjective discomfort of the clinicians. From this perspective, SAR-courses and permanent supervision would be recommended as part of basic training and continuing education.

Standardized accreditation criteria for sexology diplomas are essential to ensure parity in training rigor and adherence to contemporary evidence-based practices. Development of international standards and guidelines for educational programs can be helpful as a basis for development of regional and local training programs, and local initiatives can also inform global initiatives.

Preventive sexual health education and early counseling interventions may reduce the incidence of complex sexual dysfunctions, thereby diminishing reliance on intensive therapeutic modalities.

The fundamental right to sexual autonomy, pleasure and health equity, and the respect of human dignity and self-determination constitute an ethical imperative in sexology (Coleman et al., 2021). This discipline is inherently committed to social justice frameworks, which inform its research, education, and clinical praxis.

Despite the formal designation of sexologist in 13 European nations, institutional recognition of sexology as a distinct discipline remains inconsistent and must be adhered to. Systemic integration into national healthcare and educational policies is critical to advancing its role in sexual health promotion.

Education of policymakers on a national level and in international forms is imperative to enable them to integrate sexual health into public health initiatives broadly.

## Ethical considerations

This study did not undergo review by a human subject’s review board, as it did not meet the definition of human subject’s research under WHO’s *Standards and Operational Guidance for Ethics Review.* The survey solicited information about organizational characteristics and activities rather than personal or identifiable information about individuals and thus posed no more than minimal risk to participants. Participation was voluntary, and responses were anonymized prior to analysis. Completion of the survey was considered to imply informed consent.

## Limitations

Because this study relied on a self-selected, non-representative sample, findings should be interpreted as exploratory and descriptive. The results may be influenced by selection bias and may not reflect the views of all sexological/sexual health organizations in Europe.

This survey has focused especially on clinical education and training, but some of the programs are programs in basic/general sexology, not only for clinicians, but also include sexuality educators and sexual health promoters.

## Notes

1 Dr. Peset in Spain is included. They report a course in transgender health for medical doctors at a hospital given a doctoral degree at the end of a 100 hours educational progam

2 https://www.worldsexualhealth.net/porto-proclamation.

## Appendix

**Table:**

Examples of Programs for professionals who are licensed for clinical practice		
University courses Master’s degree		
Portugal, Porto. Master’s degree in sexology	180 ECTS	https://www.up.pt/portal/en/study/masters-degrees/courses/fpceup/27501/
Sweden, Malmo University,	120 ECTS	https://mau.se/sok-utbildning/program/sasen/#accordion-125267
University courses Master level		
Finland, Metropolia University of Applied Sciences	30 ECTS	https://www.metropolia.fi/fi/opiskelu-metropoliassa/osaamisen-taydentaminen/taydennyskoulutus/seksologia-2
Spain, University of Barcelona	24 ECTS	https://web.ub.edu/en/web/estudis/w/specificmasterdegree-202411699
Spain, University of Almeira	60 ECTS	https://www.ual.es/en/estudios/masteres/presentacion/7075
Psycho-sexology		
Croatia, Professional training in sexual psychotherapy	300 hours	https://akp.ba/en/specialist-education-in-sexual-health-and-therapy-counseling/
Germany, Munich, Certified training in sex therapy, sex counseling, sexuality and trauma	2 years	https://institut-fuer-embodiment-und-sexologie.de/seminare/seminar-sexualitaet-und-trauma/
Netherland, RINO Postgraduate sexology education	2 years	https://www.rino.nl/english
Norway Bufetat, Family office Sexology school	10 months 85 hours	Bufdir.no
Short courses		
Good Lovers Flag System, Belgium	12 hours	https://www.icrhb.org/en/projects/international-implementation-of-the-sensoa-flag-system
Norwegian Characteranalytical Institute	44 hours	https://www.karakteranalyse.no/
